# Regulated Degradation of the HIV-1 Vpu Protein through a βTrCP-Independent Pathway Limits the Release of Viral Particles

**DOI:** 10.1371/journal.ppat.0030104

**Published:** 2007-07-27

**Authors:** Emilie Estrabaud, Erwann Le Rouzic, Sandra Lopez-Vergès, Marina Morel, Nadia Belaïdouni, Richard Benarous, Catherine Transy, Clarisse Berlioz-Torrent, Florence Margottin-Goguet

**Affiliations:** 1 Institut Cochin, Université Paris Descartes, CNRS (UMR 8104), Paris, France; 2 Inserm, U567, Paris, France; Northwestern University, United States of America

## Abstract

Viral protein U (Vpu) of HIV-1 has two known functions in replication of the virus: degradation of its cellular receptor CD4 and enhancement of viral particle release. Vpu binds CD4 and simultaneously recruits the βTrCP subunit of the SCF^βTrCP^ ubiquitin ligase complex through its constitutively phosphorylated DS_52_GXXS_56_ motif. In this process, Vpu was found to escape degradation, while inhibiting the degradation of βTrCP natural targets such as β-catenin and IκBα. We further addressed this Vpu inhibitory function with respect to the degradation of Emi1 and Cdc25A, two βTrCP substrates involved in cell-cycle progression. In the course of these experiments, we underscored the importance of a novel phosphorylation site in Vpu. We show that, especially in cells arrested in early mitosis, Vpu undergoes phosphorylation of the serine 61 residue, which lies adjacent to the βTrCP-binding motif. This phosphorylation event triggers Vpu degradation by a βTrCP-independent process. Mutation of Vpu S61 in the HIV-1 provirus extends the half-life of the protein and significantly increases the release of HIV-1 particles from HeLa cells. However, the S61 determinant of regulated Vpu turnover is highly conserved within HIV-1 isolates. Altogether, our results highlight a mechanism where differential phosphorylation of Vpu determines its fate as an adaptor or as a substrate of distinct ubiquitin ligases. Conservation of the Vpu degradation determinant, despite its negative effect on virion release, argues for a role in overall HIV-1 fitness.

## Author Summary

In addition to the structural and catalytic proteins characteristic of retroviruses, HIV-1 encodes auxiliary proteins that govern virus-host interaction. One such auxiliary protein termed Vpu both enhances virion release from human cells and the degradation of CD4, the cellular surface receptor of HIV-1. The latter activity relies on a strategy frequently used by pathogens, i.e., the hijacking of the protein degradation machinery. This process involves the ubiquitin ligases that ensure the selection of the protein subsequently degraded. Vpu interacts both with an ubiquitin ligase and CD4, bridging the latter to the degradation machinery. Here, we show that in addition to subverting one particular ubiquitin ligase to trigger CD4 degradation, Vpu is itself recognized in a phosphorylation-dependent manner by a distinct ubiquitin ligase and degraded. Mutations of Vpu that confer resistance to this degradation process enhance viral particle release, whereas the determinant of Vpu turnover is paradoxically well conserved among HIV-1 isolates. These results suggest that the virus is constrained to limit its own production for long-term persistence.

## Introduction

The proteasome pathway ensures the degradation of proteins that are marked by poly-ubiquitination. This process is regulated by the E3 ubiquitin ligases that ensure the rate-limiting step of substrate selection [1]. So far, three classes of E3 ubiquitin ligases have been defined, based on the presence of a HECT, a Ring-H2-finger, and U-box domain, respectively. Within the Ring-H2-finger family are the Skp1-Cullin1-F-box proteins and the anaphase promoting complex/cyclosome complexes, which are both involved in cell-cycle progression [2]. The Skp1-Cullin1-F-box proteins display a modular organization consisting of constant core subunits (Skp1, Cullin1, Rbx1/Roc1) associated with a variable component, the F-box protein [3,4]. The F-box protein binds Skp1 through its F-box motif and recruits the substrate through a second domain. More than 70 F-box proteins have been identified in humans, each member being responsible for the degradation of a specific subset of proteins [5]. In most cases, the binding of the F-box protein requires specific phosphorylation of the substrate [6]. For example, βTrCP requires phosphorylation of the serine residues present in the consensus motif DSGΦXS, which is found in most βTrCP substrates. Phosphorylation of the βTrCP recognition motif itself is often controlled by prior phosphorylation events of the substrate protein, which allows a very tight control of the degradation process [6,7].

Viruses have developed multiple ways to subvert cell signaling pathways to replicate, disseminate, and escape from the host immune system. The diversion of the ubiquitin-mediated degradation machinery represents one such strategy, which has been first documented for the human papillomavirus E6 protein [8]. Our earlier study of the human HIV-1 viral protein U (Vpu) provided the first example reported for HIV-1 [9], and there is now a growing list of proteins from various viruses that are reported to induce the degradation of cellular targets [10,11].

Vpu is a small integral membrane protein encoded by HIV-1 and a subset of related simian immunodeficiency viruses and has no homolog in the less pathogenic HIV-2 virus [12,13]. Recent studies in macaques using simian-HIV strains have shown that Vpu plays an active role in the pathogenesis [14]. Vpu performs two major functions in HIV-1 replication [15]. First, Vpu enhances the release of retroviral particles from most human cells, including lymphocytes and primary macrophages [16–22]. This activity of Vpu, which is not observed in simian cells, is suspected to reflect its ability to counteract a host cell restriction factor specific for human cells [23–25] and may depend on Vpu binding to the host channel TASK-1 [26]. Second, Vpu induces the degradation of the CD4 viral receptor and therefore participates in the general downregulation of CD4 expression during the course of HIV infection [27]. Vpu-mediated CD4 degradation is thought to prevent CD4-Env binding in the endoplasmic reticulum in order to facilitate proper Env assembly into virions [28,29]. We earlier reported the cloning and the characterization of βTrCP as a Vpu cell partner that the viral protein connects to CD4 to induce its degradation [9]. Interaction between βTrCP and Vpu, which controls the subsequent CD4 degradation, depends on the phosphorylation of serines 52 and 56 of Vpu within its DSGΦXS βTrCP recognition motif [9,30]. In addition, the sequestration of βTrCP by Vpu has been shown to induce the stabilization of the βTrCP substrates, β-catenin and IκBα [31,32].

We and others have previously observed that Vpu remains stable during the course of CD4 degradation [9,33]. Here we report the unexpected finding that Vpu is subjected to proteasome-mediated degradation in cells arrested in early mitosis by nocodazole. We further show that this degradation process requires phosphorylation of the serine 61 residue adjacent to the βTrCP-binding motif. Therefore, differential phosphorylation of Vpu determines its fate as an adaptor or as a substrate of distinct ubiquitin ligases. The controlled degradation of Vpu, which is not mediated by βTrCP, decreases the efficiency of Vpu-mediated release of HIV-1 virions. It may thus contribute to the balance between efficient HIV-1 dissemination and preservation of the reservoir of infected cells.

## Results

### Vpu Stabilizes Cdc25A but Has No Effect on Emi1, Despite That Both Cellular Proteins Are βTrCP Substrates Involved in Cell-Cycle Progression

**Figure 1:**
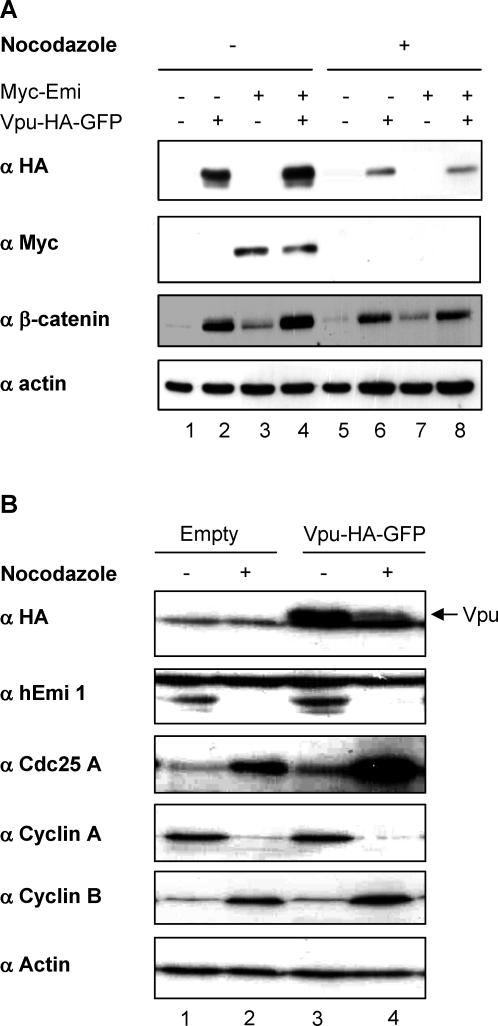
Vpu Is Degraded in Nocodazole-Treated Cells (A) Vpu does not affect Emi1 expression. HeLa cells were mock-transfected (lanes 1 and 5), or transfected with Vpu-HA-GFP alone (lanes 2 and 6), Myc-Emi 1 alone (lanes 3 and 7), or cotransfected with Vpu-HA-GFP and Myc-Emi1 (lanes 4 and 8). 24 h after transfection cells were treated 18 h with nocodazole (lanes 5–8) or DMSO (lanes 1–4). Cell lysates were separated by SDS-PAGE and analyzed by western blot using the indicated antibodies. (B) Cdc25A is stabilized in the presence of Vpu. HeLa cells expressing Vpu-HA-GFP (lanes 3 and 4) or not (lanes 1 and 2) were treated as in Figure 1A with nocodazole (lanes 2 and 4) or with DMSO alone (lanes 1 and 3). Cell lysates were separated by SDS-PAGE and analysed by western blot using the indicated antibodies. Anti-cyclin A and anti-cyclin B antibodies were used to control the efficiency of nocodazole treatment under which cyclin A but not cyclin B is degraded.

It has been well documented that sequestration of βTrCP by Vpu strongly inhibits the degradation of β-catenin and IκBα [31,32]. We investigated whether Vpu also inhibited the degradation of the cell-cycle regulators, Cdc25A and Emi1, whose degradation occurs via a βTrCP-dependent pathway in the S and pro-metaphase steps of the cell cycle, respectively [34–37]. In a first set of experiments, we used an expression construct encoding Vpu fused at its carboxyterminus to a HA-green fluorescent protein (GFP) tag that provided more sensitive and specific detection than the untagged viral protein chimera. This protein is fully functional regarding the two activities of Vpu, enhancement of particles release and CD4 degradation (Figure S1 and Text S1). As shown in Figure 1A, Myc-Emi1 was readily degraded in Hela cells arrested in early mitosis by nocodazole treatment as compared to asynchronous cells (anti-Myc panel, compare lane 3 to 7). Coexpression of Vpu did not increase the expression of Myc-Emi1 in nonsynchronized cells nor did it restore a detectable level of Myc-Emi1 in nocodazole-treated cells (Figure 1A, anti-Myc panel, compare lane 3 to 4 and lane 7 to 8). In contrast, Vpu increased the expression of endogenous β-catenin used as a control (compare lane 1 to 2 and lane 5 to 6). Confirming the lack of effect of Vpu on Emi1 accumulation, Vpu was unable to significantly stabilize endogenous Emi1, either in asynchronous or in nocodazole-treated cells (Figure 1B, anti-Emi1 panel, compare lane 1 to 3 or lane 2 to 4). Whether this lack of effect was specific for Emi1 was addressed by monitoring the accumulation of Cdc25A under the same experimental conditions. Cd525A accumulated in cells arrested in early mitosis by nocodazole treatment, as previously observed [35], and Vpu expression further increased this accumulation (Figure 1B, anti-Cdc25A panel, compare lane 1 to 2 and lanes 3 and 4). Taken together, these results indicate that Vpu differentially affects βTrCP-mediated degradation events, which occur at different phases of the cell cycle. As a possible explanation for these intriguing observations, the amount of Vpu itself varied depending on cycle progression since expression of Vpu was reduced in nocodazole-treated cells (Figure 1A, anti-HA panel, compare lane 2 to 6 and lane 4 to 8 and Figure 1B compare lane 3 to 4).

### Vpu Is Targeted by the Ubiquitin-Proteasome Pathway in Nocodazole-Treated Cells

**Figure 2:**
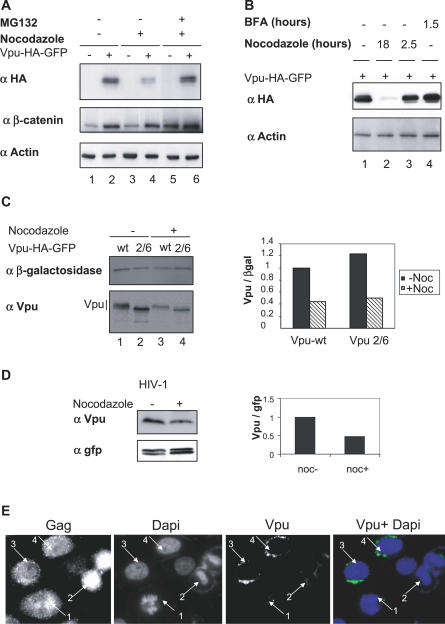
Vpu Degradation in Nocodazole-Treated Cells Is Independent of the SCF^βTrCP^ Activity (A) Vpu degradation occurs via the proteasome pathway. HeLa cells either mock-transfected (lanes 1, 3, and 5) or expressing Vpu-HA-GFP (lanes 2, 4, and 6) were left untreated or treated for 18 h with nocodazole (lanes 3–6). Where indicated, MG132 was added 6 h prior to harvest of cells. Cell lysates were analyzed by western blot using the indicated antibodies. (B) Vpu expression is not sensitive to brefeldin A or to short nocodazole treatments. HeLa cells expressing Vpu-HA-GFP were treated with nocodazole during either 18 h (lane 2) or 2.5 h (lane 3) and with BFA during 1.5 h (lane 4) or DMSO alone (lane 1). Cell lysates were separated by SDS-PAGE and analyzed by western blot using the indicated antibodies. (C) Degradadion of Vpu in nocodazole-treated cells does not require a functional βTrCP-binding site. HeLa cells were transfected with the expression vectors encoding either wild-type Vpu-HA-GFP (lanes 1 and 3) or the Vpu 2/6-HA-GFP mutant defective in βTrCP binding (lanes 2 and 4), together with a plasmid expressing Myc-βgalactosidase used as an internal reporter. Cells were left untreated or treated 18 h with nocodazole as indicated. Vpu-HA-GFP and Myc-βgalactosidase were revealed by western blot using anti-Vpu and anti-myc antibodies, respectively. The histogram represents the Vpu/βgalactosidase ratios, calculated from the respective quantified signals. (D) Vpu expression from the HIV-1 provirus is reduced in nocodazole-treated cells. HeLa cells were transfected with pNL4–3ΔVpr provirus together with a plasmid expressing GFP used as an internal control. Cells were treated with nocodazole where indicated. Vpu was detected by western blot using anti-Vpu antibodies. (E) Vpu is not detectable in infected mitotic cells. HeLa cells were transfected by the pNL4–3 provirus. 48 h after transfection, cells were fixed and analyzed by immunofluorescence using anti-Gag and anti-Vpu antibodies. DNA was revealed by DAPI staining. Arrows 1 and 2 indicate mitotic cells and arrows 3 and 4 indicate interphasic cells. wt, wild-type.

Based on previous pulse chase experiments, Vpu remains stable during the 1-h time course used to monitor Vpu-mediated CD4 degradation [33]. However, we observed in the present study that inhibition of proteasome activity by MG132 strongly increased the amount of Vpu in nocodazole-treated cells (Figure 2A, compare lane 4 to 6). Vpu expression was not affected by a shorter treatment with nocodazole used to disrupt the microtubule network without arresting cells in early mitosis (Figure 2B, compare lane 1 to 3). The efficiency of this short nocodazole treatment was checked by immunofluroescence using anti-α-tubulin antibodies (unpublished data). Vpu was previously shown to reside in the reticulum and the Golgi compartments and more recently in the recycling endosomes [25,38]. Since Golgi compartments undergo a profound redistribution during mitosis, we next asked whether decreased Vpu expression merely resulted from this disassembly process. Vpu amounts remained unchanged following treatment with brefeldin A, which promoted Golgi disassembly, as checked by immunofluorescence with an anti-rab6 antibody (Figure 2B, compare lane 1 to 4; unpublished data). Altogether, these results suggest that the mitotic arrest triggered by nocodazole, rather than an indirect effect induced by microtubules or Golgi-targeting drugs, was responsible for Vpu-induced degradation via a proteasome-dependent pathway. Furthermore, Vpu-HA-GFP was not detectable in mitotic cells by fluorescence microscopy, in contrast to the control RE-GFP protein expressed in the endoplasmic reticulum compartment (Figure S2).

We next addressed whether βTrCP was involved in Vpu degradation using a Vpu mutant defective in βTrCP binding due to the substitution of the serine residues 52 and 56 in the βTrCP recognition motif (Vpu2/6). As shown in Figure 2C, the pools of wild-type and mutant Vpu were decreased to a similar extent by nocodazole-induced mitotic arrest. That Vpu2/6 underwent degradation in early mitosis suggested that βTrCP is not responsible for Vpu degradation.

Reduction of Vpu expression in nocodazole-treated cells was further confirmed with the untagged Vpu protein expressed from the HIV-1 pNL4–3 provirus both by western blot (Figure 2D) and by immunofluorescence (Figure 2E). In the latter experiment, anti-Gag labeling identified infected cells, and DAPI staining was used to distinguish interphasic from mitotic cells. As shown in Figure 2E, Vpu was readily detected in interphasic cells by a perinuclear staining (cells 3 and 4) whereas no signal was observed in mitotic cells (cells 1 and 2).

### Identification of a New Phosphorylation Determinant in Vpu Involved in the Viral Protein Turnover

**Figure 3:**
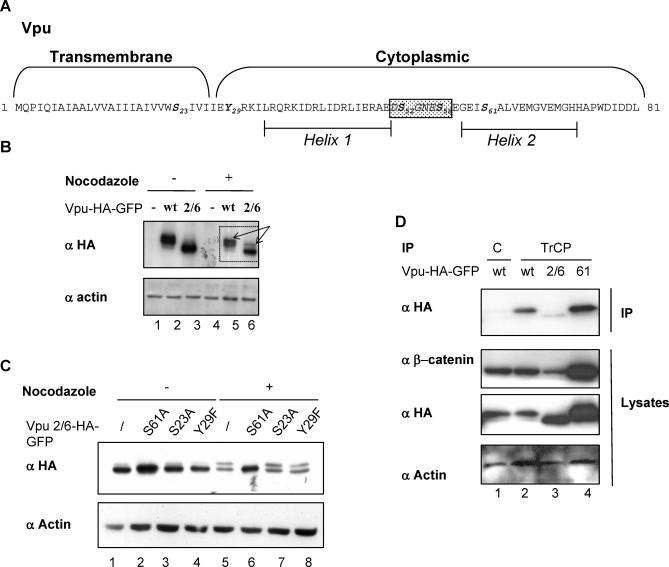
Identification of a New Phosphorylation Determinant in Vpu (A) Schematic representation of the Vpu sequence from the HIV-1 NL4–3 strain. Potential phosphorylation residues are indicated in bold. These include two serine residues (S23 and S61) and one tyrosine (Y29), in addition to the S52 and S56 present in the boxed βTrCP-binding motif. (B) A new form of Vpu and Vpu 2/6 is detected in nocodazole-treated cells. HeLa cells were mock-transfected (lanes 1 and 4), transfected by Vpu-HA-GFP (lanes 2 and 5), or by Vpu 2/6-HA-GFP (lanes 3 and 6). 24 h after transfection, cells were treated 18 h with nocodazole (lanes 4–6) or with DMSO (lanes 1–3). Cell lysates were separated by SDS-PAGE and analyzed by western blot using the indicated antibodies. (C) Vpu is phosphorylated on serine 61 in nocodazole-treated cells. HeLa cells were transfected with the indicated Vpu variants: Vpu2/6-HA-GFP (lanes 1 and 5), Vpu2/6 S61A (lanes 2 and 6), Vpu 2/6 S23A (lanes 3 and 7), and Vpu 2/6 Y29F (lanes 4 and 8). Cells were treated during 18 h with nocodazole (lanes 5–8) or with DMSO (lanes 1–4). Cell lysates were separated by SDS-PAGE and analyzed by western blot using the indicated antibodies. (D) VpuS61A conserves its ability to interact with βTrCP. Lysates from HeLa cells expressing Vpu-HA-GFP (lanes 1 and 2), Vpu 2/6-HA-GFP (lane 3), or Vpu S61A-HA-GFP (lane 4) were immunoprecipitated with anti-βTrCP (IP TrCP, lanes 2–4) or with control antibodies (IP C, lane 1). Immunoprecipitates and cell lysates were separated by SDS-PAGE and analyzed by western blot using the indicated antibodies.

A slow migrating form of Vpu and of Vpu2/6 was apparent when lysates from nocodazole-treated cells were subjected to extended electrophoresis (Figure 3B, arrows). Treatment with alkaline phosphatase of Vpu and Vpu2/6 immunoprecipitates from mitotic cell extracts suppressed the slow migrating Vpu species (Figure S3). This indicated that a new phosphorylation event of Vpu was taking place in mitosis. We individually mutated the three potential phosphorylation sites present in Vpu outside of the βTrCP recognition motif, i.e., S23, Y29, and S61 (Figure 3A). Substitution of serine 61 into alanine within the Vpu2/6 mutant precluded the appearance of the slow migrating form seen in nocodazole-treated cells, whereas mutations of S23 and Y29 had no effect (Figure 3C, lanes 5–8). Mutation of serine 61 in the context of wild-type Vpu led to identical results (unpublished data).

**Figure 4:**
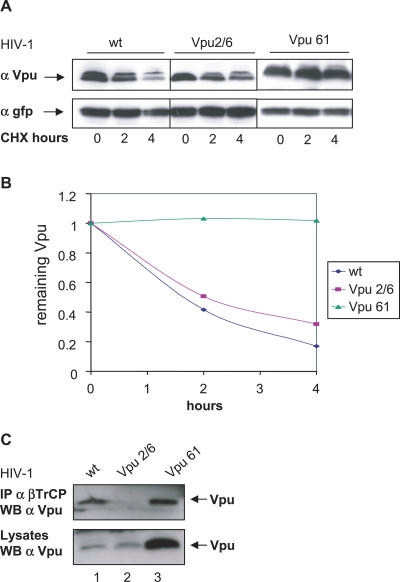
Mutation of Vpu S61 in the HIV-1 Virus Markedly Extends the Viral Protein Half-Life While Preserving Its Interaction with βTrCP (A) HeLa cells were transfected with 0.5 μg of HIV-1 proviral DNA corresponding to pNL4–3 wild-type, pNL4–3 Vpu 2/6, or pNL4–3 Vpu S61Q together with 0.05 μg of GFP expression vector used as a control for the transfection. Cells were treated with cycloheximide and harvested at the indicated time points. Cell lysates were separated by SDS-PAGE and analyzed by western blot for GFP and Vpu expression. (B) Quantification of Vpu expression shown in (A). After quantification, the signals obtained in (A) were used to calculate the Vpu/GFP expression ratios at the indicated time points after cycloheximide addition. (C) The S61 Vpu mutant conserves binding to βTrCP in cells transfected with HIV-1 proviral DNAs. HeLa cells were transfected with 0.5 μg of HIV-1 proviral DNAs encoding the indicated Vpu proteins. Cell lysates were immunoprecipitated with anti-βTrCP antibodies and analyzed for Vpu expression (top) or directly analyzed with Vpu antibodies (bottom).

We hypothesized that Vpu phosphorylation on serine 61 provided the signal that triggered Vpu degradation in mitotic cells. In support of this hypothesis, the Vpu S61A mutant strongly accumulated in cells as compared to the wild-type protein (Figure 3D, compare lane 2 to 4). In addition, Vpu S61A coimmunoprecipitated very efficiently with endogenous βTrCP (Figure 3D, first panel, lane 4), and accordingly, strongly stabilized β-catenin (Figure 3D, second panel, lane 4). As expected, Vpu2/6, used as a control, did not interact with βTrCP and did not stabilize β-catenin (Figure 3D, lane 3). These results were further confirmed in the context of the HIV-1 NL4–3 virus. To avoid a change in the viral envelope overlapping sequence, the Vpu serine 61 was changed into a glutamine. Introduction of Vpu S61Q mutation in the NL4–3 provirus extended the half-life of the Vpu protein; whereas, as previously reported [33], S52A and S56A mutations hardly affected the degradation rate of the protein (Figure 4A and 4B). Consistent with our previous observations, interaction with βTrCP was not affected by the S61Q mutation (Figure 4C, lane 3). Altogether, these results support the conclusion that degradation of Vpu occurs via a βTrCP-independent pathway and is triggered by phosphorylation of the S61 residue.

### Mutation of Vpu Serine 61 Enhances the Release of HIV-1 Particles

**Figure 5:**
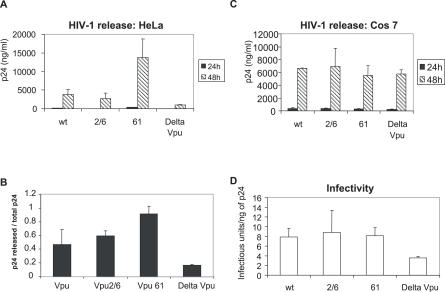
Release of HIV-1 Particles Is Enhanced by Vpu S61 Mutation (A) Vpu S61 mutation enhances HIV-1 release from the Vpu-sensitive HeLa cells. HeLa cells were transfected with 0.5 μg of the indicated HIV-1 proviral DNAs. Capsid p24 antigen was quantified in culture cell supernatants 24 and 48 h after transfection. (B) The tranfected cells described in (A) were lyzed 48 h post-transfection and analyzed for their capsid p24 content. The graph represents the ratios of released p24 released over total p24 amount (released + internal), 48 h post-transfection. (C) Vpu S61 mutation has no effect on HIV-1 release from the Vpu-insensitive Cos 7 cells. Release was studied in Cos 7 cells as indicated in (A). (D) Vpu S61 mutation does not alter HIV-1 infectivity. The indicated viruses were produced in Hela cells (A) and their infectivity was assayed in Magi cells. Results are expressed as the ratios of infectious particles in Magi cells per ng of p24 capsid protein in the input virus.

Vpu-mediated CD4 degradation was not affected by mutation of Vpu serine 61 (unpublished data) as was expected from the preserved interaction of the mutant with βTrCP (Figures 3D and 4C). We therefore investigated whether this mutation affected the other known Vpu activity, namely, the enhancement of viral particle release that we assayed by monitoring the extracellular content in mature p24 capsid. As shown in Figure 5A, HIV-1 encoding the S61 Vpu mutant was released much more abundantly from HeLa cells than the wild-type virus. As reported previously, S52A and S56A mutations of Vpu had no effect on virus release whereas the virus NL4–3ΔVpu was less efficiently released (Figure 5A). Quantitative analysis of the ratio of released capsid protein over total amount of capsid indicated that the S61Q Vpu mutant increased the viral release process rather than the expression of the viral capsid (Figure 5B). To assess the specificity of these effects with respect to the human-restricted activity of Vpu, we further studied HIV-1 particle release from African green monkey Cos 7 cells, where Vpu has been shown to be dispensable for efficient virion release [19,24]. Wild-type and Vpu-mutated HIV-1 were released from these cells with similar efficiencies (Figure 5C). We further analyzed the infectivity of the viral particles from HeLa cell supernatants (from experiment 5A). Vpu S61 mutation did not affect the infectivity of the viral particles (Figure 5D). Altogether, these results indicate that HIV-1 particle release is increased when the Vpu degradation process is impaired by the mutation of serine 61.

## Discussion

The major finding of our study is the identification of a new phosphorylation determinant in the HIV-1 Vpu protein, which controls the efficiency of HIV-1 particle release by regulating the turnover of the viral protein.

### Vpu Inhibitory Function of the SCF^βTrCP^ E3 Ubiquitin Ligase

Our initial goal was to study the potentially adverse effects of Vpu expression on the timely regulated degradation of βTrCP substrates involved in cell-cycle progression. To our surprise, Emi1 escaped the Vpu-mediated stabilization observed for the constitutively degraded βTrCP substrate, β-catenin, and for the cell-cycle regulator Cdc25A, normally degraded during the S phase. This paradox may be reconciled by our further demonstration of a regulated degradation process of Vpu itself, which predominantly occurs in early mitosis. Indeed, one simple explanation is that the pool of Vpu drops before the cell cycle reaches the phase where Emi1 degradation takes place, namely at the G2-M transition. Another nonexclusive possibility is the existence of a βTrCP pool dedicated to Emi1 degradation [39], which is nonaccessible to Vpu.

### Vpu as an Adaptor or a Substrate of E3 Ubiquitin Ligases

**Figure 6:**
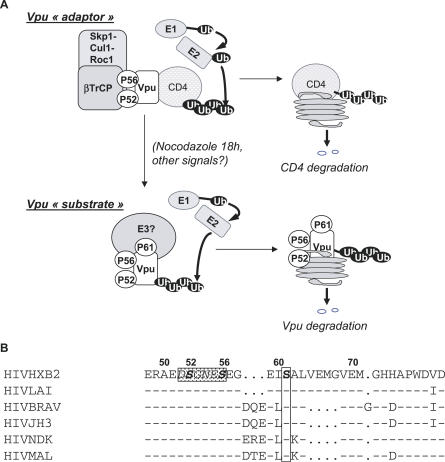
Model for Vpu Degradation (A) Vpu as an adaptor of the SCF^βTrCP^ complex or as a substrate targeted to the proteasome via a βTrCP-independent pathway. Vpu is constitutively phosphorylated on the serine 52 and 56 residues present in the βTrCP-binding site. Phosphorylation of these serines is essential for Vpu binding to βTrCP and for CD4 degradation. By bridging βTrCP, a subunit of the SCF^βTrCP^ complex to CD4, Vpu induces proteasome-mediated degradation of CD4. In this case, Vpu acts as an adaptor of the βTrCP complex, now able to target CD4. Here, we underscore an additional phosphorylation event taking place at the S61 residue. This phosphorylation site is critical for Vpu turnover and for the control of virus release. In this other setting, Vpu behaves as a substrate of an E3 ubiquitin ligase distinct from the βTrCP complex. (B) The determinant of regulated Vpu turnover is highly conserved within HIV-1 isolates. Among the Vpu sequences from 100 strains of HIV-1 group M analyzed by McCormick-Davis and coworkers (2000) [46], 77% show a serine residue, either at position 61 or at position 64, i.e., 4 and 7 residues downstream of the βTrCP-binding motif (in the rectangle). In addition, this serine is always preceded by the EI or EL di-residue. Only six representative Vpu sequences have been chosen in our alignment to highlight this conservation. “−”, aa identical to HIVHXB2 and “.”, gaps introduced for optimal alignment of sequences.

We have shown that the phosphorylation of Vpu serine 61, which lies adjacent to the βTrCP recognition motif, is the triggering event for subsequent Vpu degradation. Accordingly, mutation of Vpu serine 61 into a non-phosphorylable residue stabilized Vpu, expressed either alone or in the context of the HIV-1. Two lines of evidence indicate that Vpu degradation occurs via a βTrCP-independent pathway: (i) The Vpu mutant, which fails to recruit βTrCP due to substitutions in its binding motif, was degraded in mitosis-arrested cells to the same extent as the wild-type protein; (ii) conversely, the S61 Vpu mutant, which still binds βTrCP, showed a much slower turnover than the wild-type protein. In turn, this implies that Vpu is recognized as a substrate by an E3 ubiquitin ligase other than βTrCP, as illustrated in the model presented in Figure 6. Vpu has been shown to be constitutively phosphorylated by casein kinase II on serine 52 and serine 56 of the βTrCP-binding motif [40]. As a result, Vpu binds βTrCP and acts as an adaptor between the SCF^βTrCP^ and CD4, leading to the degradation of the HIV-1 receptor [9]. In contrast, the new phosphorylation event underscored here, which takes place on the serine 61, creates a binding site for an E3 ubiquitin ligase, which in turn targets Vpu for proteasome-mediated degradation. Phosphorylation of the serine 61 residue does not depend on prior phosphorylation of the βTrCP-binding motif since it still occurs when residues 52 and 56 are mutated. However, binding to the two E3 ubiquitin ligases might be mutually exclusive, considering the proximity between their respective binding sites. How the viral protein escapes the degradation via the SCF^βTrCP^ ubiquitin ligase while it is recognized as a substrate by a second ubiquitin ligase remains an open question. It should be emphasized that such an issue holds for other viral proteins that subvert E3 ubiquitin ligases. For example, HPV E6 and E7 proteins show the same dual fate as Vpu: they act as adaptors of an E3 ubiquitin ligase to direct the abnormal degradation of a cellular protein for the benefit of the virus [11] and as substrates of a probably distinct E3 ubiquitin ligase [41–44]. In the same line, the HIV-1 Vpr protein, which recruits the DDB1-Cul4A E3 ubiquitin ligase to induce cell-cycle arrest as shown in our recent study [45], appears to be stabilized when incorporated in this complex (unpublished data).

### Limiting the Release of Viral Particles

We show here that the slower turnover of Vpu resulting from S61 substitution increases the release of HIV-1 virions, hence suggesting a selective advantage. This prompted us to examine the degree of conservation of the corresponding Vpu residue among HIV-1 isolates. To address this point, we took advantage of the previous analysis performed by McCormick-Davis and coworkers (2000) on the Vpu sequences from 100 strains of HIV-1 group M from diverse geographical regions [46]. Remarkably, 77% of these strains showed a serine residue, either at position 61 or at position 64, i.e., 4 and 7 residues downstream of the βTrCP-binding motif (Figure 6B and unpublished data). Of note, this spacing difference corresponds to one helix turn, which suggests that the relevant serine residue is presented on the same face of the Vpu α-helix. This indicates that within the M group of HIV-1, the evolutionary trend is toward the conservation of a regulated Vpu degradation process. HIV-1 replicates in both quiescent and dividing cells. In the latter, it might be advantageous for the virus to fine-tune virion production according to cell-cycle progression. This may in turn reflect that long-term persistence of the virus requires the optimal balance between efficient viral production and host cell viability. In light of our present study, the regulated degradation process of Vpu is expected to be lost in the viral strains naturally mutated at the relevant phosphorylation site. Whether these strains show higher pathogenicity will be interesting to address for our better understanding of the constraints that govern the relation between HIV-1 and the human organism.

## Materials and Methods

### Plasmid constructs.

pcDNA3 Vpu-HA-GFP and Vpu2/6-HA-GFP were described in Besnard-Guerin et al., 2004 [32]. Mutations of S23, S61, and Y29 in pcDNA3 Vpu-HA-GFP and pcDNA3 Vpu2/6-HA-GFP were introduced by site-directed mutagenesis with *Pfu* Turbo polymerase (Stratagene, http://www.stratagene.com/) using the following set of forward primers where substituted codons are underlined: Vpu S23A: 5′-GCAATAGTTGTGTGGGCCATAGTAATCATAG-3′; Vpu Y29A: 5′-CCATAGTAATCATAGAATTTAGGAAAATATTAAGAC-3′; Vpu S61A: 5′-GAGAGTGAAGGAGAAATAGCAGCACTTGTGGAGATGG-3′).

### Proviral DNAs.

The pNL4–3 HIV1 clones as well as its Vpu- or Vpr-deficient derivative were gifts from Dr. K. Strebel. Mutations of the *vpu* gene were done using a pUC19 shuttle vector containing the Eco RI-Nhe I fragment (5743–7251 [47]) by directed mutagenesis with *Pfu* Turbo polymerase (Stratagene). Vpu2/6 and Vpu S61 variants were created using the following set of forward primers, where substituted codons are underlined: Vpu2/6: 5′-GAAAGAGCAGAAGACAATGGCAATGAGAATGAAGGAGAAGTA-3′; VpuS61:5′- GAGTGAAGGAGAAGTACAAGCACTTGTGGAGATG-3′.

All constructs were verified by nucleotide sequencing with a DYEnamic ET Terminator kit (Amersham, http://www.gehealthcare.com/) and a Genetic Analyzer (Applied Biosystems, http://www.appliedbiosystems.com/).

### Cell culture, transfection procedures, and treatments.

HeLa, P4R5 MAGI cells, and Cos 7 cells were maintained in DMEM supplemented with glutamine and 10% fetal calf serum. Plasmid transfections were performed using Fugene 6 reagent (Roche, http://www.roche.com/). Nocodazole (Sigma, http://www.sigmaaldrich.com/) was used at the final concentration of 450 ng/ml. Brefeldin A (Calbiochem, http://www.calbiochem.com/) was used at 5 μM during 1.5 h. Cells were treated with MG132 (Sigma) at 20 μM during 6 h.

### Immunoprecipitation, western blot procedures, and antibodies.

Cells grown in 10-cm dishes were lysed in SD buffer (50 mM Tris-HCl [pH 7.5], 150 mM NaCl, 0.5% Triton X100) for 30 min at 4 °C. Cell lysates were clarified by centrifugation and incubated with primary antibodies for 2 h at 4 °C or overnight using anti-βTrCP antibodies. Protein G-sepharose from Sigma (20 μl of 50% slurry in SD buffer) was added and the samples were further rocked at 4 °C for 30 min. After four washes in lysis buffer, immunoprecipated proteins were recovered by incubation in Laemmli buffer (Sigma) at 95 °C for 5 min and separated by SDS-PAGE electrophoresis. Following transfer onto PVDF membranes, proteins were revealed by immunoblot analysis using a chemiluminescent procedure (ECL, GE Healthcare, http://www.gehealthcare.com/). Signals were acquired by a LAS-3000 apparatus (Fujifilm, http://www.fujifilm.com/) for further quantification, using the provided software Multigauge. Monoclonal antibodies directed at the HA (3F10) and MYC (9E10) and GFP tags were obtained from Roche. Anti-actin (I-19), anti-cyclin A (H-432), anti-cyclin B1 (H-433), and monoclonal anti-Cdc25A (F-6) were purchased from Santa Cruz Biotechnology (http://www.scbt.com/); monoclonal anti-β-catenin was obtained from Transduction Laboratories (http://www.bdbiosciences.com/). Anti-Vpu was purified from the antiserum (NIH 969) as described below. Anti-βTrCP and anti-Emi1 are gifts from K. Strebel and P. Jackson, respectively. The p24 HIV-1 capsid protein was revealed using a rabbit anti-p24 polyclonal antibody (ARP366, National Institute for Biological Standards and Control, Hertfordshire, United Kingdom).

### Purification of Vpu antiserum.

Recombinant GST fused to the cytoplasmic domain of Vpu was separated by SDS-PAGE electrophoresis. Following transfer onto PVDF membrane, the Vpu antiserum (NIH 969) was incubated with the membrane in TBS buffer (50 mM Tris, 150 mM NaCl) containing 0.05% Tween 20 and 5% milk overnight at 4 °C. After four washes in TBS 0.05% Tween, the membrane was incubated 10 min at 4 °C in PBS. The purified antibodies were eluted in 0.2 M glycine/HCl buffer at room temperature. The acidic pH was neutralized by adding 2 M Tris.

### Immunofluorescence assays.

For p24 capsid and Vpu staining, HeLa cells were seeded in 6-well plates onto glass cover slips at a density of 2 × 10^5^ cells/well, and transfected with 1 μg of pNL4–3. 48 h post-transfection, cells were washed in PBS and fixed for 1 h in 4% paraformaldehyde, quenched for 10 min in PBS /0.1 M glycine, and then permeabilized for 10 min in IF buffer (PBS, BSA 1 mg/ml, 0.01% Triton X100). Cells were then incubated with purified anti-Vpu (1:500) and anti-p24 capsid (25A Hybridolab, 1:500, http://www.pasteur.fr/) antibodies during 1 h, washed in IF buffer, and incubated for 30 min with Cya2-labeled anti-rabbit (1:300) and Cya3-labeled (1:750) anti-mouse antibodies (Jackson ImmunoResearch, http://www.jacksonimmuno.com/). After washes in IF buffer and mounting in medium containing DAPI (Vectashield, http://www.vectorlabs.com/), cells were examined by direct fluorescent microscopy.

### HIV-1 production assay.

HeLa and Cos 7 cells (2 × 10^5^ cells) were transfected with 0.5 μg of different pNL4–3 proviral plasmids (wild-type, ΔVpu, Vpu 2/6, Vpu61) using Fugene 6 reagent (Roche). Cell supernatants were collected 24 and 48 h post-transfection and quantified for HIV-1 p24 antigen using an HIV-1 p24 antigen assay (Beckman Coulter, http://www.beckmancoulter.com/). Cell supernatants harvested 48 h post-transfection were also used to infect P4R5 Magi indicator cells. Protein cell lysates were separated by SDS/PAGE and analyzed by western blot.

## Supporting Information

Figure S1 Vpu-HA-GFP Is Functional Regarding the Two Main Activities of Vpu (A) Vpu-HA-GFP enhances viral particle release. HeLa cells were transfected with 0.5 μg of HIV-1 proviral DNAs (wild-type or ΔVpu) and 0.2 μg of Vpu-HA-GFP expressing vector (or the corresponding empty vector) where indicated. p24 antigen was quantified in culture cell supernatants 24 and 48 h after transfection.
(B) Vpu-HA-GFP downregulates CD4 expression.
P4–2 cells were transfected with Vpu-HA-GFP or Vpu 2/6-HA-GFP. Cells were analyzed by FACS for the expression of both CD4 and Vpu. After transfection of Vpu-HA-GFP, 46.1% (23 + 23.1) of the cells express Vpu; half of them are CD4-negative (FACS panel, left). Therefore, about 50% of the cells expressing Vpu are CD4-negative (see histogram). After transfection of Vpu2/6-HA-GFP, 49% (46.2 + 2.8) of the cells do express Vpu; 2.8% of them are CD4-negative (FACS panel, right). Therefore, about 5.7% of the cells expressing Vpu2/6-HA-GFP are CD4-negative (see histogram). Expression of both Vpu-HA-GFP and Vpu2/6-HA-GFP was checked by western blot.
(54 KB PDF)

Figure S2 Vpu-HA-GFP Is Not Detected in Mitotic Cells HeLa cells were transfected with Vpu-HA-GFP or the control RE-GFP protein expressed in endoplasmic reticulum compartment. 36 h after transfection, cells were fixed and analyzed by fluorescence. DNA was revealed by staining with DAPI. Stars indicate cells in mitosis.
(86 KB PDF)

Figure S3 Vpu Is Phosphorylated Outside the DSGXXS Motif HeLa cells were mock-transfected (lanes 1 and 6), transfected with Vpu-HA-GFP (lanes 2, 3, 7, 8), or with Vpu 2/6-HA-GFP (lanes 4, 5, 9, 10). 24 h after transfection, cells were treated with nocadazole (lanes 6–10) or DMSO alone (lanes 1–5) during 18 h. Cell lysates were immunoprecipitated using an anti-HA antibody. Immunoprecipitates were left untreated (lanes 1, 2, 4, 6, 7, and 9) or treated with alkaline phosphatase (lanes 3, 5, 8, and 10) and analyzed by western blot using anti-GFP antibodies.
(53 KB PDF)

Text S1 Supplementary Materials and Methods (20 KB DOC)

### Accession Numbers

The GenBank (http://www.ncbi.nlm.nih.gov/Genbank/) accession numbers for the proteins and genes studied here are EMI1 (NM_012177) and HIV-1 molecular clone pNL4–3 (AF324493).

## Abbreviations

GFP: green fluorescent protein
Vpu: viral protein U
