# Supplementary figures and images for: Regulated Degradation of the HIV-1 Vpu Protein through a βTrCP-Independent Pathway Limits the Release of Viral Particles
Source: PLoS Pathog. 2007 Jul 27;3(7):e104. doi: 10.1371/journal.ppat.0030104 (PMC1933454; doi:10.1371/journal.ppat.0030104)

A

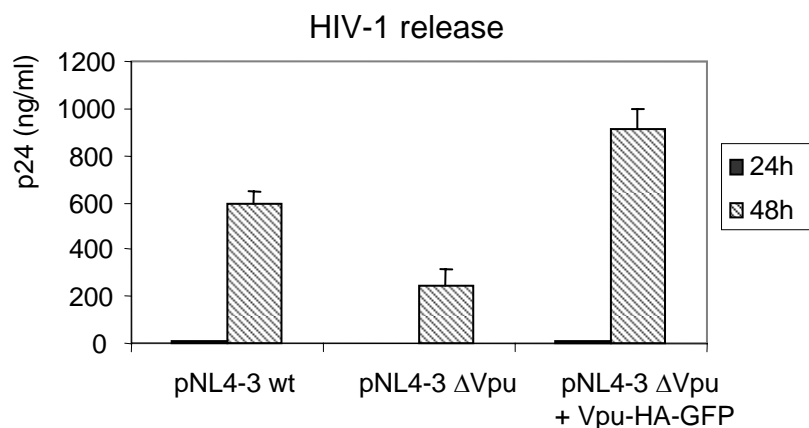

B

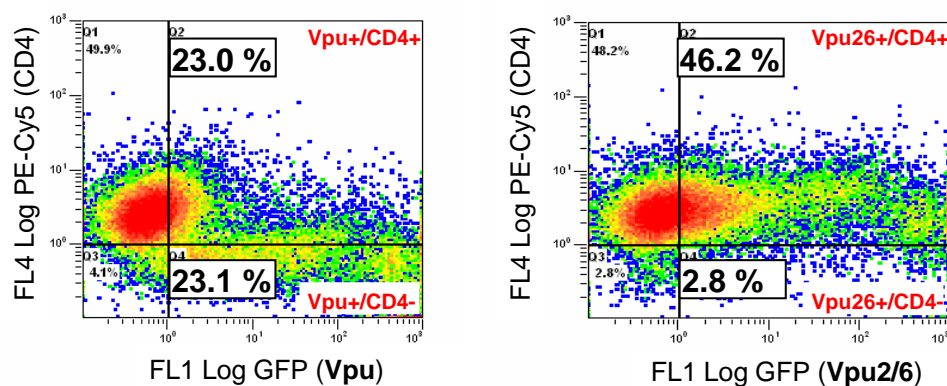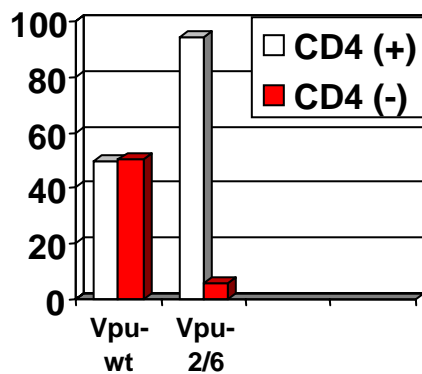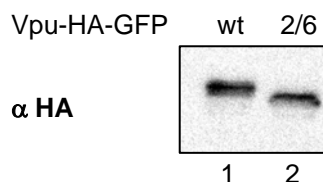

Estrabaud et al.  
Supplementary Figure 1

Supplement: Figure S1 — (A) Vpu-HA-GFP enhances viral particle release. HeLa cells were transfected with 0.5 μg of HIV-1 proviral DNAs (wild-type or ΔVpu) and 0.2 μg of Vpu-HA-GFP expressing vector (or the corresponding empty vector) where indicated. p24 antigen was quantified in culture cell supernatants 24 and 48 h after transfection. (B) Vpu-HA-GFP downregulates CD4 expression. P4–2 cells were transfected with Vpu-HA-GFP or Vpu 2/6-HA-GFP. Cells were analyzed by FACS for the expression of both CD4 and Vpu. After transfection of Vpu-HA-GFP, 46.1% (23 + 23.1) of the cells express Vpu; half of them are CD4-negative (FACS panel, left). Therefore, about 50% of the cells expressing Vpu are CD4-negative (see histogram). After transfection of Vpu2/6-HA-GFP, 49% (46.2 + 2.8) of the cells do express Vpu; 2.8% of them are CD4-negative (FACS panel, right). Therefore, about 5.7% of the cells expressing Vpu2/6-HA-GFP are CD4-negative (see histogram). Expression of both Vpu-HA-GFP and Vpu2/6-HA-GFP was checked by western blot. (54 KB PDF) [file ppat.0030104.sg001.pdf]

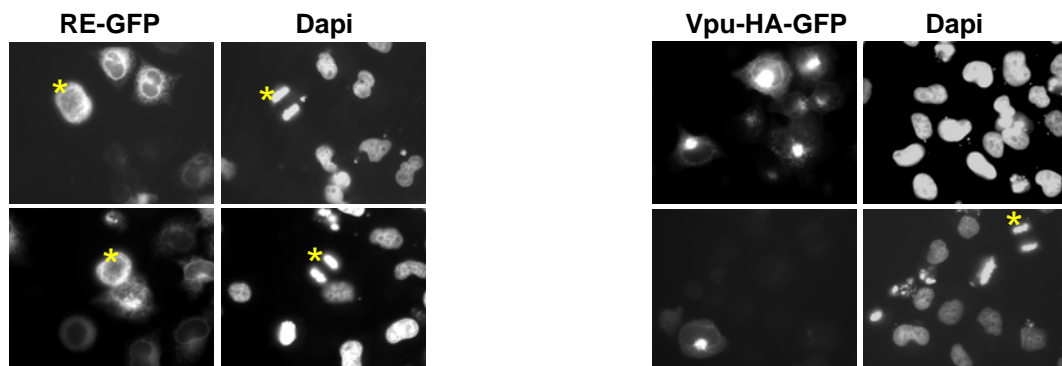

Estrabaud et al.  
Supplementary Figure 2

Supplement: Figure S2 — HeLa cells were transfected with Vpu-HA-GFP or the control RE-GFP protein expressed in endoplasmic reticulum compartment. 36 h after transfection, cells were fixed and analyzed by fluorescence. DNA was revealed by staining with DAPI. Stars indicate cells in mitosis. (86 KB PDF) [file ppat.0030104.sg002.pdf]

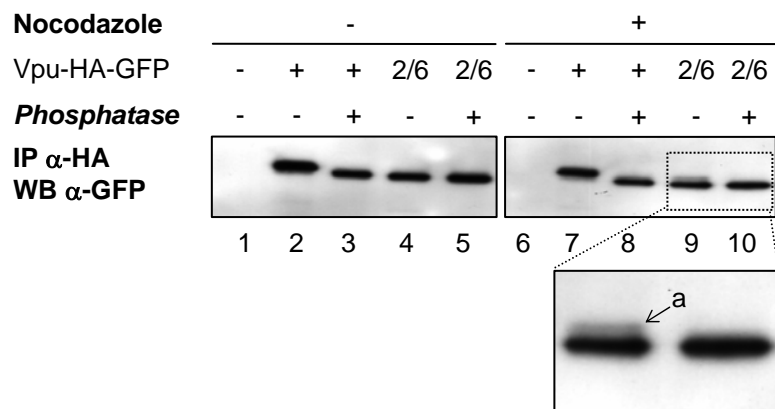

Estrabaud et al.  
Supplementary Figure 3

Supplement: Figure S3 — HeLa cells were mock-transfected (lanes 1 and 6), transfected with Vpu-HA-GFP (lanes 2, 3, 7, 8), or with Vpu 2/6-HA-GFP (lanes 4, 5, 9, 10). 24 h after transfection, cells were treated with nocadazole (lanes 6–10) or DMSO alone (lanes 1–5) during 18 h. Cell lysates were immunoprecipitated using an anti-HA antibody. Immunoprecipitates were left untreated (lanes 1, 2, 4, 6, 7, and 9) or treated with alkaline phosphatase (lanes 3, 5, 8, and 10) and analyzed by western blot using anti-GFP antibodies. (53 KB PDF) [file ppat.0030104.sg003.pdf]
