# Supplementary material for: Regulated Degradation of the HIV-1 Vpu Protein through a βTrCP-Independent Pathway Limits the Release of Viral Particles
Source: PLoS Pathog. 2007 Jul 27;3(7):e104. doi: 10.1371/journal.ppat.0030104 (PMC1933454; doi:10.1371/journal.ppat.0030104)
Supplement: Text S1 — (20 KB DOC) [file ppat.0030104.sd001.doc]

**Supplementary materials and methods**

**Immunodetection of CD4 by flow-cytometry:**

HeLa P4-2 cells were harvested 36h hours after transfection with Vpu-HA-GFP or Vpu 2/6 -HA-GFP. Cells were incubated on ice 30 min in PBS/ 2 % FBS. Cell pellets were then incubated with anti-CD4-PE-Cy5 (BD Pharmingen) (1:2 dilution in PBS 2% FBS during 45 min on ice in the dark. After 2 washes in PBS 2% FBS, cell pellets were re-suspended in PBS (10^6^ cells/ml) and analyzed using a Cytomics FC500 cell analyzer (Beckman Coulter).

**Detection of a phosphorylation event by treatment with a phosphatase enzyme.**

Cells transfected by Vpu-HA-GFP and Vpu 2/6 -HA-GFP were harversted and lysed 48 hours after transfection. Vpu-HA-GFP was immunoprecipitated using anti-HA antibodies. Immunoprecitates were divided in two fractions. The first fraction was treated with alkaline phosphatase (CIP, Biolabs) during 45 min, and the second fraction was left untreated. Immunoprecipitates were separated by SDS-PAGE and analyzed by western blot.
